# Tackling the non-communicable disease epidemic: a framework for policy action in low- and middle-income countries

**DOI:** 10.11604/pamj.2024.47.82.41089

**Published:** 2024-02-26

**Authors:** Mark Fordjour Owusu, Joseph Adu, Sebastian Gyamfi, Ebenezer Martin-Yeboah, Benjamin Ansah Dortey

**Affiliations:** 1 Higher Education Development Center, University of Otago, Christchurch Central City, Christchurch 8011, New Zealand,; 2 Department of Health and Rehabilitation Sciences, Western University, Ontario, Canada,; 3 Faculty of Nursing, University of Windsor, Windsor, Ontario, Canada,; 4 Lawson Health Research Institute, London, Ontario, Canada,; 5 Department of Health Information Science, Western University, London, Ontario, Canada,; 6 Trauma and Specialist Hospital, Winneba, Ghana

**Keywords:** Health policy frameworks, non-communicable disease, prevention, control, lower-income countries, middle-income countries, high-income countries

## Abstract

Health policy frameworks for the prevention and control of non-communicable diseases have largely been developed for application in high-income countries. Limited attention has been given to the policy exigencies in lower- and middle-income countries where the impacts of these conditions have been most severe, and further clarification of the policy requirements for effective prevention is needed. This paper presents a policy approach to prevention that, although relevant to high-income countries, recognizes the peculiar situation of low-and middle-income countries. Rather than a narrow emphasis on the implementation of piecemeal interventions, this paper encourages policymakers to utilize a framework of four embedded policy levels, namely health services, risk factors, environmental, and global policies. For a better understanding of the non-communicable disease challenge from a policy standpoint, it is proposed that a policy framework that recognizes responsible health services, addresses key risk factors, tackles underlying health determinants, and implements global non-communicable disease conventions, offers the best leverage for prevention.

## Perspectives

Current global trends show that non-communicable diseases (NCDs) constitute a significant percentage of disease burden [1] but the policy framework for their prevention and control presents a daunting challenge, particularly in Low-and Middle-Income Countries (LMICs). In the past, communicable diseases such as malaria, HIV/AIDS, and diarrheal conditions were responsible for a high proportion of the global burden of disease. In 1990, communicable, nutritional, and neonatal conditions accounted for 47% of the global burden of disease, with this decreasing to 35% while NCDs increased to 54% by 2010 [2]. Currently, it is estimated that NCDs cause 41 million global deaths annually, which correspond to 74% of all mortalities, with 15 million of these involving the active working population between the ages of 30 and 69 years, and 85% of this taking place in LMICs [3]. In addition, burden of disease analyses shows that a significant proportion of global disabilities are attributable to NCDs. This is because estimates show that Years Lived with Disability (YLD) have increased with rising NCD trends, from 537.6 million in 1990 to 764.8 million in 2013 and this has been compounded by increasing incidence of risk factors, particularly tobacco smoking, excessive salt and alcohol intake, and physical inactivity [1].

The high NCD burden and mortalities have significant socioeconomic impacts on the global community particularly on LMICs. Evidence shows that NCD deaths lead to poverty, especially in LMICs where the dependency ratio is high [3]. As NCDs kill people in their active working years, these affect families, plunging them further into poverty. Not only this, since these conditions have a long duration, treatment costs are high and may impact negatively on populations who pay out-of-pocket. In Nigeria for example, the cost of NCDs represents 24% of annual household expenditure [4]. Thus, these conditions exacerbate the poverty situation as there is a cyclical relationship between NCDs and poverty [5]. This is because people with NCD complications may not be able to work to earn money, thus pushing them further into poverty, which in turn makes it difficult for them to maintain treatment. The economic repercussions of NCDs go beyond individuals and families. They affect general economic activity and growth. In India, Mahal and colleagues [6] reported that if not for NCDs, GDP would have been between 4-10% higher in 2004. Not only this, NCDs have been known to reduce wages and increase absenteeism, deplete human resources, decrease retirement ages, and contribute to higher rates of unemployment and economic inactivity [7]. Thus, in low-resource settings such as those in most African countries, uncontrolled NCDs could further deepen underdevelopment, as scarce resources may be used to treat people with one or more NCDs.

In general, these economic impacts are linked to psychosocial burdens in LMICs, where NCDs impoverish families who then abandon relatives, with research establishing a correlation between NCDs and social isolation in some countries [8]. Again, NCDs have been known to lead to stigmatization and marginalization. In Ghana, evidence shows that women living with cancer and diabetes may not only be shunned but in some rural areas, those with uncontrolled diabetes characterized by extreme weight loss face stigma identical to that of those with HIV/AIDS [8]. The challenge, therefore, is to reverse the increasing mortality and morbidity across the globe, particularly in LMICs, where a significant proportion of the global poor live, and access to health services remains inadequate by introducing appropriate policies [2,3].

Although the international community provides support, national governments are encouraged to make NCD control and prevention a priority. However, it appears very little has been achieved in developing effective policies to prevent and control NCDs in many countries. In general, policy response from the international community has been slow across the globe [9]. While progress has been made in NCD policy response in some countries, most of these remain fragmented. It appears several countries have been quick to ratify global protocols without attuning contextual factors to aid implementation efforts, a phenomenon which can partly be attributed to an inadequate understanding of the complex framework that characterizes NCD control and prevention. Consequently, policy response from the global community does not correspond to the burden of disease attributable to NCDs [7,9].

Current frameworks for understanding NCD policy approaches have focused on the potential impacts of specific healthcare interventions although the policy demands for effective control transcend the adoption of piecemeal interventions. The models and frameworks put forward have focused on behavioral health promotion for the prevention of NCDs [10]; a combination of interventions such as enhancement of the environment, policy change, and community collaboration [11]; and health determinants [12]. Apart from the fact that these frameworks are intervention-driven, they largely came from high-income country (HIC) contexts. While these frameworks provide insights for populating ideas about the NCD policy enterprise, the nature of health systems and the level of development in LMICs require broader policy thinking. Therefore, it can be argued that such frameworks may not work well in LIMCs given the plethora of contextual policy challenges. On the other hand, given the challenges that HICs are also facing in fighting the NCD epidemic (as NCDs are a global challenge), it would be too simplistic to argue that policies framed in developed settings may not work in LMICs. Hence, one could argue that it is often not so much that HICs are transferring what (or a framing that) worked in their setting to LMICs, but that they are transferring what (or a framing that) did not work to LMICs. With these issues in mind, our aim here was to present a framework, that, although focusing on LMICs, is nevertheless applicable to HICs in the prevention and control of NCDs. Consequently, the main objective of this treatise was to suggest a structure for framing policy understanding for the prevention and control of NCDs, particularly in LMICs.

**Key issues in non-communicable disease prevention policies:** we believe that NCDs are complex to manage and require a whole-of-society approach. A policy approach must, therefore, encompass legislation and frameworks to enhance understanding at various levels. Drawing from the literature, we provide a list of issues we consider relevant for a better understanding of NCD prevention and control from a policy standpoint.

Traditionally, health services have been targeted at professionals such as physicians and nurses [13]. However, for effective NCD control, there is the need for policies with emphasis on responsive services that address demand and enable patients to make critical and informed choices rather than on partial interventions with limited impact on systems behaviour. This is crucial because it is the behaviour of the system that triggers expected changes and not partial interventions. Therefore, a policy framework for NCD prevention must recognize the role patients play in the determination and utilization of appropriate services. Also, services must be provided cost-effectively. This is important because of the limited resources in LMICs. With evidence showing that only a few LMICs have adopted cost-effective treatment services [14], the need for a policy framework that focuses on the adoption of such interventions in secondary prevention to increase access to NCD services is paramount. Thus, the policy should recognize that beyond doctors and nurses, an array of other health workers at primary, secondary, and tertiary levels need to be engaged in NCD control services. Also, the role of significant others who participate in providing direct and indirect care must be factored into the policy framework.

Non-communicable disease policies must tackle shared risk factors which include physical inactivity, poor diet, tobacco use, and excessive alcohol intake [3]. For risk factor policies to achieve desired outcomes, the process through which they are developed is crucial. Recent studies in LMICs have revealed several challenges such as poor intersectoral coordination, limited institutional capacity, and funding gaps in risk factor policy development [7,9]. With the implementation of policies depending on the formulation process [9], it is important to ensure effective linkages between policy development and implementation. Since risk factor policies involve several stakeholders with conflicting views and interests, the key is to address the interests of all relevant stakeholders at the policy development stage to garner support and engender buy-in during implementation. This must closely be aligned to the adoption of cost-effective policy interventions for the management of risk factors. This is particularly important given the financial challenges in LMICs. Although LMICs generally constitute fertile grounds for adopting cost-effective interventions for the management of risk factors, they are hardly used in these countries [14].

The socio-economic determinants of health are a fundamental aspect of an effective NCD policy structure as they represent a formidable approach to prevention. These determinants include income levels, opportunities for education, occupation, employment status, access to food, utility services, housing, and social cohesion. Evidence reveals that educational status, income levels, and occupation are associated with cardiovascular health; with good urban design, streetlights, and recreation parks contributing to increased physical activity levels [14,15]. Policies on these determinants may not address NCDs directly but have the potential to cause a change or control the elements in the physical and social environment leading to a long-lasting impact on the overall health of populations. To be effective, therefore, NCD policy frameworks must reflect a whole-of-society approach addressing challenges in education, poverty, underdevelopment, and deprivation.

A policy structure for NCD management must take into consideration the role of global protocols, conventions, declarations, and action plans in complementing national policy efforts. A significant number of countries have ratified international protocols and declarations to manage NCDs. International NCD policy frameworks such as the Framework Convention on Tobacco Control and the Global Strategy on Diet, Physical Activity, and Health have been instrumental in NCD plans and policies in several countries. However, the crucial role of international protocols and conventions in addressing the NCD challenge received a major boost following the UN High-Level Meeting and the Moscow Health Ministers´ Conference in 2011. These landmark events marked a clear pathway to be followed in the NCD fight, with emphasis on LMICs. Among other considerations, the meetings affirmed that NCDs had reached epidemic proportions and called for national governments to act. Global frameworks and policies should, therefore, be an integral part of NCD frameworks and policies. To ensure proactive actions, policy actors who attended these conventions must take lead roles in resource mobilization, advocacy, and implementation oversights. Not only these, internationally accepted evidence-based, cost-effective interventions must be adopted at different levels of prevention to support context-specific approaches. For example, the WHO best buys have proved to be relevant in preventing NCDs in various contexts [16] and must form part of a potpourri of policy options implemented for NCD prevention.

**Figure 1:**
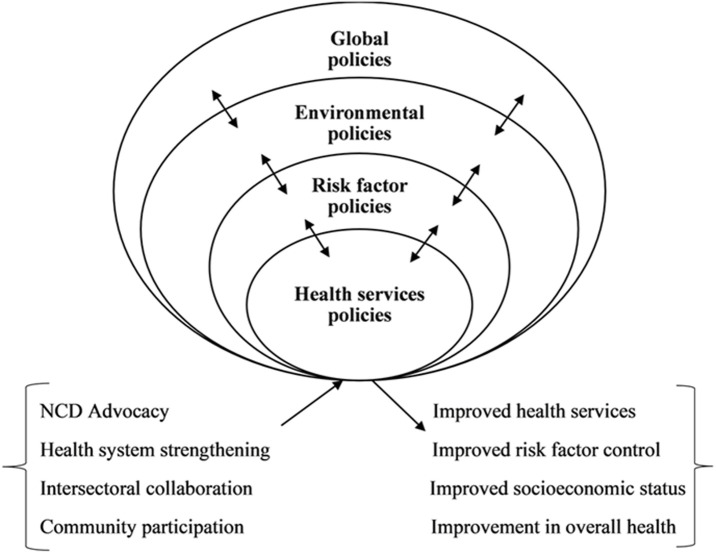
policy framework for non-communicable disease prevention

**A policy framework for non-communicable disease prevention:** Figure 1 represents a broad policy approach for controlling and preventing NCDs based on the key issues discussed above. Policy levels bidirectionally influence each other (shown by arrows) to produce desired impacts. The framework encourages national governments to approach the NCD challenge in four embedded policy levels in addition to the adoption of policy support mechanisms. Below, we use a few examples to foreground our discussion and to show how the above framework could be applied in the prevention and control of NCDs. The policy levels include: i) health services policies; ii) risk factor policies; iii) environmental policies; iv) global and international policies.

**Level 1- health services policies:** as access to NCD services is poor globally and particularly in LMICs [14], policy frameworks must reflect and address the service needs of individuals and communities at risk or suffering from one or more NCDs. The literature is replete with examples of poor access and utilization of NCD services across countries due to the absence of clear-cut health services policies. In Ghana for example, challenges in the public sector mean that patients in rural areas find it difficult to access health services as prices are high in the private facilities, with access to NCD medicines a major concern [7]. Screening, diagnosis, and treatment services remain a big challenge in Nepal [17], while in China, as high as 37% of patients experience catastrophic health expenditure following a single stroke episode and fall below the poverty line [18]. These examples call for policy frameworks that utilize cost-effective NCD services. The provision of drug therapy, counseling services, and vaccination against human papillomavirus are among the WHO cost-effective best buy interventions recommended for effective treatment [16]. The problem, however, is that many LMICs have been very slow to adopt these cost-effective interventions. For example, research supports the effectiveness of beta-blockers and aspirin to reduce vascular problems separately, but with even better results when taken in combined doses [19]. Evidence confirms the availability and affordability of multidrug regimens for the treatment of NCDs although few LMICs have adopted such interventions [14]. Given that cost remains a major challenge in accessing some of these services in LMICs, the adoption of best-buy interventions could be a masterstroke as they have been known to be particularly cost-effective [16]. Thus, poor access may not only be attributed to physical distance but lack of funds to purchase existing diagnostic tools for which deliberate cost reduction measures by governments are critical.

**Level 2- risk factor policies:** in general, a significant proportion of NCD deaths would be averted if risk factors were addressed [3]. It is, therefore, not enough to provide NCD services. A policy framework must also tackle shared NCD risk factors. Nesting health services policies into risk factor policies (as shown in our framework) will not only make it possible for individuals suffering from NCDs to obtain the needed services but also ensure that those susceptible to one or more risk factors access appropriate care. For risk factor policies, the policy process is vitally important. There are several examples of poor processes affecting the policy scenario for NCDs in many countries. Juma *et al*. [20] reported that in Cameroon, South Africa, Malawi, and Nigeria, funding gaps in NCD policy development meant that some non-governmental and civil society organizations used their resources to participate in policy meetings. Since this was not sustainable in the long term, some policies were developed without input from several stakeholders, negatively impacting their implementation. Oladepo *et al*. [21] reported that apart from tobacco policies, stakeholder engagement was low for other risk factor policies in Nigeria. In South Africa, salt legislation was developed without the inclusion of several institutions in the food industry [20]. Poor policy leadership regarding risk factors has also affected NCD policy processes in several countries including Malawi, where a non-governmental organization, Drug Fight Malawi, had to spearhead the development of the alcohol policy while the National Authority for the Campaign against Alcohol and Drug Abuse (NACADA) played a cardinal role in the development of alcohol policies in Kenya. In addition, there is a need to ensure appropriate stakeholder analysis in the policy development process and engagement for specific NCD types [9].

Apart from issues related to the policy process, adopting cost-effective policy approaches for risk factor control remains important but is usually neglected in LMICs. Tobacco taxation, a key WHO best-buy intervention for the prevention of tobacco use, is one perfect example of this. In some HICs, this intervention has been used in the past to effectively combat relapse and increase smoking cessation, with research showing that the price elasticity is almost double in LMICs [14]. The implication is that increasing the price of tobacco by a certain percentage in LMICs will lead to twice the reduction in smoking in relation to HICs. While this should be a good motivation, LMICs have been very slow in adopting tobacco taxation as the proportion of taxes in cigarette prices is very low in these countries compared to HICs. For example, although Ghana has been fairly successful in reducing the prevalence of smoking by developing tobacco policies, the total excise tax on tobacco products is only 16.1% of the average retail price compared to the WHO-set target of 70% [22]. The NCD policy framework must, therefore, detail appropriate risk factor interventions based on available scientific evidence such as the ones put forward in the WHO best-buys.

**Level 3- environmental policies:** socio-economic factors are usually overlooked in NCD policy discussions in both HICs and LMICs. Studies have shown that higher education, employment, and adequate income are significantly correlated with higher population health outcomes although this remains a major challenge even in HICs [14]. In New Zealand, a HIC, the indigenous Maori population has limited access to key health determinants such as education, employment, and housing leading to poor health outcomes among these communities [23]. Distribution of mortalities and disabilities, poor secondary prevention, unhealthy NCD-related behaviours, NCD-related stress, and poor access to drugs have all been implicated in the poor management of the determinants of health [7]. In LMICs, NCD efforts continue to focus on clinical interventions with limited emphasis on wider environmental factors that affect health [9]. There are serious challenges associated with accessing clean water, food, electricity, and recreational facilities all of which affect health. Therefore, the key is to adopt policy approaches that view the NCD challenge as a societal problem that goes beyond the adoption of health interventions to address challenges in education, deprivation, environmental issues, and improvement in the overall standard of living.

**Level 4- global and international policies:** the last level in our framework relates to international policies. In general, several countries recognize the role these global conventions, protocols, action plans, and interventions play in their NCD policy efforts. Nonetheless, there is still work to be done in terms of the implementation of these protocols and conventions in LMICs. For example, in a comprehensive analysis, Allen *et al*. [24] examined the implementation of global NCD policies in 151 countries and found a mean implementation score of 49.3%, with Costa Rica and Iran having the joint highest score of 86.1%. However, scores for some countries were as low as 5.5%, indicating that while many countries ratify these international policies, a significant number are unable to implement them. Although the reasons differ from country to country, a lack of financial resources, human resource challenges, poor intersectoral collaboration, over-emphasis on infectious diseases, and inadequate data are among the factors identified for poor implementation of these conventions and policies [9]. Within the context of LMICs, international protocols may be described as mere window dressing with limited impact on NCD prevention. The fact is that these conventions have failed to address the negative effects of globalization on the NCD efforts of LMICs, with Glasgow and Schrecker [25] attributing this to the neoliberal idea of ‘choice', an underpinning policy phenomenon in HICs that is being trumpeted in LMICs and which may be seen as a typical case of a policy idea not working in HICs but being transferred to LMICs. The point here is that the assumption that people can make healthy choices for themselves (the neoliberal idea of choice) has proved unsuitable in the NCD policy efforts of both HICs and LMICs because of the low level of control that people have regarding their exposure to risk factors. This ‘choice’ thinking influences health promotion plans in many countries resulting in an overemphasis on interventions such as education and counseling which, although helpful, nevertheless, have little effect on population health. Thus, for LMICs to realize the benefits of international conventions and frameworks, there is a need for stronger internal regulatory mechanisms that adapt these protocols to national exigencies and contexts.

**Policy support mechanisms:** the policy framework presented here recognizes the relevance of several policy support mechanisms. These include NCD advocacy, health system strengthening, intersectoral collaboration, and community participation. A continuous program of NCD advocacy has been suggested to keep people abreast of lifestyle changes necessary for risk factor control [26]. Activities may include policy monitoring and stakeholder accountability, policy dialogue, social rallies and campaigns, and research. Intersectoral action is not only necessary during the development and implementation of NCD policies but also a crucial aspect in the management of the social determinants of health. Poor collaboration between the health sector and other sectors such as agriculture, trade and industry, education, and urban planning have jeopardized gains in many countries [7,9,20-22].

The literature is full of examples of successful community-participated bottom-up NCD programs in HICs which are being adopted successfully in LMICs [26,27]. Evidence shows that the successful implementation of health policies is partly influenced by the degree of commitment and knowledge of local inhabitants [27]. This is because when local people 'own' NCD programs, they are enthusiastic about their implementation. Regrettably, decision-making in many countries has remained centralized amidst decentralized health systems, a phenomenon attributed to limited local capacity and failure to integrate public health issues into reform efforts [28]. Local authorities in LMICs must, therefore, build capacity and highlight evidence of the public health relevance of various interventions by participating in designing, monitoring, and evaluating health initiatives. Due to weak health systems, a total system strengthening must underpin policy efforts with an emphasis on governance, funding, human resources, health information systems, delivery of appropriate services, and technological considerations [29]. The framework shows that successful implementation of subnational, national, and international policies with appropriate support mechanisms in an integrated way has the potential to lead to improved health services and outcomes.

## Conclusion

This paper shows that the current fragmented policy approaches being utilized, although useful, may not be effective in addressing the complex issues involved in NCD prevention and control. Instead, a broad framework detailing four embedded policy levels is suggested. This policy approach encourages health managers to pay attention to responsive NCD health services and adopt effective policy processes and appropriate interventions in addressing risk factors. Additionally, the framework recognizes policies on the social determinants of health as a key component of NCD prevention. Since local and national policies are shaped by the distribution of power and resources at the global level, there is a need to recognize the role of international NCD protocols and conventions in prevention and control efforts. The framework advocates for appropriate mechanisms to support policies and programs. These include a continuous program of health system strengthening and advocacy, community participation, and intersectoral collaboration. While this framework provides useful insights into NCD policy understanding, it may be somewhat idealistic to prescribe a 'single-jacket' approach for addressing the NCD quandary since this depends, to a large extent, on policy actors in any given nation. Consequently, appreciating the specific NCD policy context, processes, and actors of each nation and how these interact to shape the policy scenario is key. This notwithstanding, it is argued that an understanding of the NCD challenge from a whole-of-society perspective offers the best leverage to stem the tide of a group of diseases which, put together, contribute close to 80% of the disease burden in the world.

## References

[1] Institute for Health Metrics and Evaluation 2019 Global Burden of Disease Study.

[2] Murray CJ, Vos T, Lozano R, Neghavi M, Flaxman AD, Michaud C (2012). Disability-adjusted Life Years for 291 Diseases and Injuries in 21 Regions, 1990-2010: A Systematic Analysis for the Global Burden of Disease Study 2010. Lancet.

[3] World Health Organization (2023). Noncommunicable diseases.

[4] Odunyemi A, Rahman T, Alam K (2023). Economic burden of non-communicable diseases on households in Nigeria: evidence from the Nigeria living standard survey 2018-19. BMC Public Health.

[5] Peters DH, Garg A, Bloom G, Walker DG, Brieger WR, Hafizur Rahman M (2008). Poverty and access to health care in developing countries. Ann N Y Acad Sci.

[6] Mahal A, Karan A, Engelgau M (2010). The economic implications of non-communicable disease for India. HNP Discussion Paper.

[7] Owusu MF (2019). Effective management of non-communicable diseases in Ghana: the case of hypertension and diabetes mellitus [Doctoral Thesis, University of Canterbury]. The University of Canterbury Thesis Repository.

[8] Aikins AD (2006). Reframing applied disease stigma research: a multilevel analysis of diabetes stigma in Ghana. Journal of Community & Applied Social Psychology.

[9] Owusu MF, Basu A, Barnett P (2019). Hypertension and Diabetes Management: A Policy Perspective from Ghana. J Health Organ Manag.

[10] US Public Health Service (1994). For a healthy nation: Return on investments in public health.

[11] Grizzell J (2009). High Reach/Low-Cost Health Agenda Programming.

[12] Hamilton N, Bhatti T (1996). Population health promotion: An integrated model of population health and health promotion: Health Canada.

[13] Mohanty A, Kabi A, Mohanty AP (2019). Health problems in healthcare workers: A review. J Family Med Prim Care.

[14] Gaziano TA, Pagidipati N (2013). Scaling up Chronic Disease Prevention Interventions in Lower-and Middle-Income Countries. Annu Rev Public Health.

[15] Devarajan R, Prabhakaran D, Goenka S (2020). Built environment for physical activity-An urban barometer, surveillance, and monitoring. Obes Rev.

[16] World Health Organization (2017). Tackling NCDs: 'best buys' and other recommended interventions for the prevention and control of noncommunicable diseases. World Health Organization.

[17] Khanal S, Veerman L, Nissen L, Hollingworth S (2017). Use of healthcare services by patients with non-communicable diseases in Nepal: a qualitative study with healthcare providers, Journal of clinical and diagnostic research. J Clin Diagn Res.

[18] Lau WC, Froehlich JB, Jewell ES, Montgomery DG, Eng KM, Shields TA (2013). Impact of adding aspirin to beta-blocker and statin in high-risk patients undergoing major vascular surgery. Ann Vasc Surg.

[19] Bazrafshan N, Lotfi MM (2016). A multi-objective multi-drug model for cancer chemotherapy treatment planning: A cost-effective approach to designing clinical trials. Computers & Chemical Engineering.

[20] Juma PA, Mohamed SF, Mwagomba BLM, Ndinda C, Mapa-Tassou C, Oluwasanu M (2018). Non-communicable disease prevention policy process in five African countries authors. BMC Public Health.

[21] Oladepo O, Oluwasanu M, Abeona O (2018). Analysis of Tobacco Control Policies in Nigeria: Historical Development and Application of Multi-sectoral Action. BMC Public Health.

[22] Sarpong G (2018). Teenage smokers: How Ghana´s low excise tax on cigarette encourage the deadly habit.

[23] Ministry of Health New Zealand (2015). Tatau Kahukura: Māori Health Chart Book 2015.

[24] Allen LN, Nicholson BD, Yeung BY, Goiana-da-Silva F (2020). Implementation of non-communicable disease policies: a geopolitical analysis of 151 countries. Lancet Glob Health.

[25] Glasgow S, Schrecker T (2016). The Double Burden of Neoliberalism? Noncommunicable Disease Policies and the Global Political Economy of Risk. Health Place.

[26] Maharani A (2022). Participation in community-based healthcare interventions and non-communicable diseases early detection of general population in Indonesia. SSM Popul Health.

[27] Bullock HL, Lavis JN (2019). Understanding the supports needed for policy implementation: a comparative analysis of the placement of intermediaries across three mental health systems. Health Res Policy Syst.

[28] Lele G (2023). Concurrency as crisis decision-making governance: Lessons from Indonesia's response to the COVID-19 pandemic. Regional & Federal Studies.

[29] United States Agency for International Development (2021). USAID´s vision for health system strengthening 2030.

